# Genomic and functional characterization of a *bla*_NDM-1_ and *tmexCD2-toprJ2* co-producing *Klebsiella quasipneumoniae* (KL159/ST1929) reveals plasmid-mediated suppression of virulence

**DOI:** 10.1128/spectrum.02401-25

**Published:** 2026-03-17

**Authors:** Feng Nie, Tianyu Zou, Ruihang Luo, Maosen Liu, Hongyi Lai, Yanghua Xiao, Jingwen Zhang, Yang Liu, Wei Zhang, Ping Li, Tianxin Xiang

**Affiliations:** 1 Jiangxi Provincial Key Laboratory of Respiratory Diseases, Jiangxi Institute of Respiratory Diseases, The Department of Respiratory and Critical Care Medicine, Jiangxi Clinical Research Center for Respiratory Diseases, The First Affiliated Hospital, Jiangxi Medical College, Nanchang University, Nanchang, China; 2 China-Japan Friendship Jiangxi Hospital, National Regional Center for Respiratory Medicine 47861, Nanchang, Jiangxi, China; 3 First Clinical Medical College of Nanchang University, Nanchang University 89668, Nanchang, Jiangxi, China; 4 Department of Clinical Microbiology, The First Affiliated Hospital, Jiangxi Medical College, Nanchang University, Nanchang, China; 5 Jiangxi Medical Center for Critical Public Health Events, The First Affiliated Hospital of Nanchang University 117970, Nanchang, Jiangxi, China; Department of Pathology, City of Hope, Duarte, California, USA

**Keywords:** *Klebsiella quasipneumoniae*, *bla*_NDM-1_, tmexCD2-toprJ2, plasmid curing, antimicrobial resistance, virulence suppression, whole-genome sequencing

## Abstract

*Klebsiella quasipneumoniae* has emerged as a multidrug-resistant (MDR) pathogen of growing clinical concern, driven by its capacity to acquire mobile genetic elements encoding carbapenemases and novel resistance determinants. This study aimed to characterize the genomic architecture, resistance profile, and phenotypic impact of plasmids in a clinical *K. quasipneumoniae* strain co-producing *bla*_NDM-1_ and the tigecycline efflux pump gene cluster *tmexCD2-toprJ2*. Whole-genome sequencing and plasmid reconstruction were performed for strain KP10883 (KL159/ST1929). Antimicrobial susceptibility testing, plasmid curing, biofilm quantification, serum resistance assays, human lung epithelial cell infection assays, and *Galleria mellonella* infection models were used to evaluate the functional consequences of plasmid carriage. KP10883 harbored two functionally distinct plasmids: pKP10883-1, an IncU conjugative plasmid carrying *bla*_NDM-1_ and *tmexCD2-toprJ2*, conferring broad-spectrum antibiotic resistance, and pKP10883-2, a nonconjugative IncFIB/IncFII plasmid encoding stability and adaptation modules without classical resistance genes. Curing of pKP10883-1 resulted in complete loss of resistance to β-lactams, carbapenems, and tigecycline, confirming plasmid-mediated resistance. Paradoxically, plasmid loss led to increased biofilm formation, enhanced serum resistance, greater epithelial cell adhesion and cytotoxicity, and higher virulence in *G. mellonella*, indicating suppression of chromosomal virulence by plasmid-borne regulatory elements. This study reveals a plasmid-mediated trade-off between antimicrobial resistance and virulence in *K. quasipneumoniae*. The functional complementarity of pKP10883-1 and pKP10883-2 supports their stable coexistence and reflects a broader adaptive strategy balancing drug resistance with pathogenic potential. These findings underscore the importance of considering the regulatory impact of MDR plasmids on host virulence in developing strategies against emerging MDR pathogens.

**IMPORTANCE:**

The emergence of multidrug-resistant (MDR) *Klebsiella quasipneumoniae* poses an escalating threat to clinical treatment and infection control. This study provides the first comprehensive genomic and functional characterization of a clinical *K. quasipneumoniae* strain co-harboring *bla*_NDM-1_ and the tigecycline efflux pump cluster *tmexCD2-toprJ2*. By integrating genomic, phenotypic, and virulence analyses, we reveal a paradoxical trade-off in which plasmid acquisition confers extensive antibiotic resistance while suppressing host virulence. These findings uncover a novel plasmid-mediated regulatory mechanism balancing bacterial survival and pathogenicity. Understanding this interplay between resistance and virulence expands the current knowledge of MDR plasmid biology and provides valuable insights into how resistance evolution may reshape bacterial pathogenic potential—information critical for surveillance, therapeutic development, and containment of emerging high-risk *Klebsiella* lineages.

## OBSERVATION

Klebsiella pneumoniae is a versatile pathogen that colonizes humans, animals, and environmental reservoirs (1). The *K. pneumoniae* species complex (*KpSC*) comprises *K. pneumoniae sensu stricto* (KpI), *K. quasipneumoniae* (KpII), and *K. variicola* (KpIII), which differ in the genomic background, ecological adaptation, and antimicrobial resistance profiles (2). Within KpII-B, *K. quasipneumoniae subsp. similipneumoniae* (*Kqss*) has long been underrecognized due to phenotypic similarity to classical *K. pneumoniae* (2, 3). With the wider adoption of whole-genome sequencing (WGS), *Kqss* is increasingly implicated as a reservoir for high-risk resistance determinants and as a contributor to hospital-associated persistence and transmission (4, 5). Reports documenting the acquisition of *bla*_NDM_ or *bla*_KPC_ by *Kqss*, persistent colonization of plumbing, and plasmid remodeling via transposition and homologous recombination point to an underappreciated clinical threat (6, 7). Nevertheless, functional insights into how multidrug resistance intersects with virulence in *Kqss* remain limited. During routine genomic surveillance, we identified a multidrug-resistant *Kqss* isolate, KP10883 (KL159/ST1929), co-harboring *bla*_NDM-1_ and the RND-type efflux pump cluster *tmexCD2-toprJ2*. We combined hybrid genome assembly with functional assays to define its genetic architecture and to test the hypothesis that large resistance plasmids modulate the virulence output.

**Fig 1:**
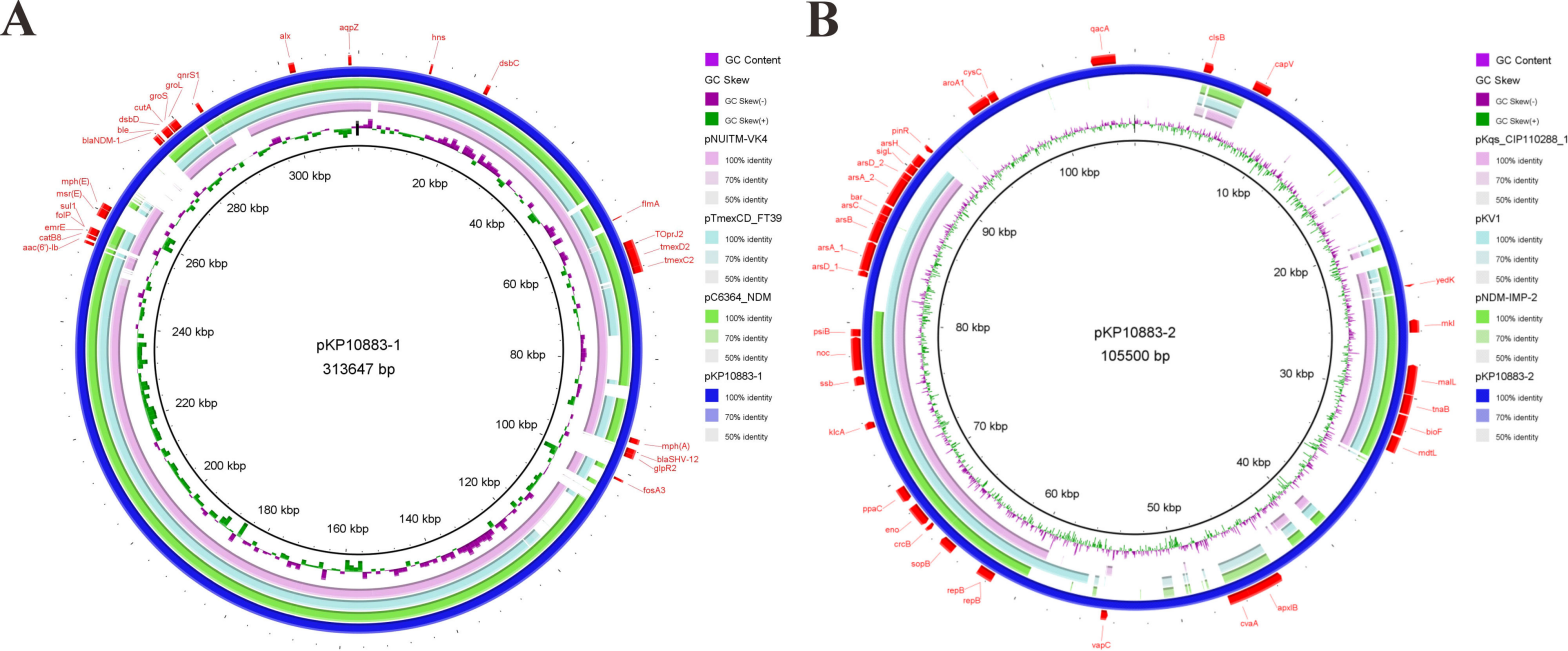
Circular map of plasmid pKP10883-1 (313,647 bp) (**A**) and circular map of plasmid pKP10883-2 (105,500 bp) (**B**). (**A**) The outermost ring shows annotated antimicrobial resistance genes, including *bla*_NDM-1_, *bla*_SHV-12_, *qnrS1*, *mph(A*), *mph(E*), *msr(E*), *fosA3*, *catB8*, and *sul1*, conferring resistance to β-lactams, quinolones, fosfomycin, macrolides, chloramphenicol, and sulfonamides. Inner rings represent sequence alignments with reference plasmids pNUITM-VK4 (*Klebsiella quasipneumoniae*, 84% coverage, 99.99% identity, AP025165.1), pTmexCD_FT39 (*K. pneumoniae*, 84% coverage, 99.97% identity, CP132737.1), and pC6364_NDM (*K. pneumoniae*, 83% coverage, 99.99% identity, MZ532980.1), highlighting conserved multidrug resistance backbones with high nucleotide identity. The innermost tracks depict GC content (purple) and GC skew (green/pink), with deviations indicating potential horizontally acquired regions. The high similarity to multiple globally disseminated MDR plasmids suggests pKP10883-1 may play a key role in the spread of carbapenem and extensively drug-resistant determinants among Klebsiella species. (**B**) The outermost ring displays genes related to heavy metal resistance (*arsA/B/C/D/H*), toxin-antitoxin modules (*vapC* and *cvaA/cvaB*), and plasmid stability/maintenance (*repB*, *sopB*, *noc*, and *ssb*). Alignment rings show sequence similarity with pKqs_CIP110288_1, pKV1, and pNDM-IMP-2, revealing conserved plasmid backbones with evidence of recombination events. The innermost rings represent GC content and GC skew. Although pKP10883-2 lacks acquired antibiotic resistance genes, it carries IncFII_1_pKP91 and IncFIB(K)_1_Kpn3 replicons, suggesting its potential role as a mobilizable plasmid that may facilitate co-transfer of other resistance plasmids.

Hybrid assembly yielded a 5.17-Mb chromosome and two plasmids: a 313,647-bp IncU megaplasmid, pKP10883-1, and a 105,500-bp IncFII/IncFIB plasmid, pKP10883-2. A circular representation of pKP10883-1 highlights a complex organization with discrete resistance modules (Fig. 1A). pKP10883-1 is a novel IncU-type conjugative plasmid that simultaneously carries *bla*_NDM-1_ and *tmexCD2-toprJ2*, conferring resistance to carbapenems and tigecycline. The *bla*_NDM-1_ gene is embedded in a canonical NDM-1 context flanked by ISAba30, bleMBL, and ISKpn19, highly conserved relative to previously reported NDM-1 plasmids (e.g., pC6364_NDM). In contrast, the *tmexCD2-toprJ2* cluster is bracketed by ISKpn28 and IS881, consistent with composite transposon-mediated acquisition. Comparative alignment showed that pKP10883-1 shares 84% query coverage and >99.97% identity with known MDR plasmids, including pNUITM-VK4 (AP025165.1), pTmexCD_FT39 (CP132737.1), and pC6364_NDM (MZ532980.1). BLAST analysis revealed >99% identity between the *tmexCD2-toprJ2* region and the corresponding region of pTmexCD_FT39, while the *bla*_NDM-1_ module closely matched that of pC6364_NDM. These data indicate that pKP10883-1 is a mosaic backbone integrating 2 major MDR modules through recombination of distinct plasmid lineages. To our knowledge, this is the first report of an IncU-type plasmid co-harboring *bla*_NDM-1_ and *tmexCD2-toprJ2* in *K. quasipneumoniae*, underscoring its potential role in disseminating carbapenem and tigecycline resistance in clinical settings.

A circular map of pKP10883-2 (Fig. 1B) illustrates a genetic architecture compatible with plasmid maintenance and environmental fitness. The plasmid encodes heavy-metal resistance determinants (*arsA/B/C/D/H*), toxin-antitoxin systems (*vapC and cvaA/cvaB*), and replication/partitioning genes (*repB, sopB, ssb, and noc*). Additional metabolic and regulatory genes*—aroA1*, *crcB*, *ppaC*, *bioF*, and *mdlT*—are distributed throughout the plasmid. Comparative analysis showed high similarity to pKqs_CIP110288_1, pKV1, and pNDM-IMP-2, supporting a mosaic backbone shaped by multiple recombination events. GC content and GC skew profiles suggest acquisition of genomic islands via horizontal gene transfer. Notably, pKP10883-2 carries IncFII_1_pKP91 and IncFIB(K)_1_Kpn3 replicons typical of conjugative plasmids, implying potential mobility. Although pKP10883-2 lacks classical antibiotic resistance genes (ARGs), its metal-resistance clusters and toxin-antitoxin systems likely enhance stability under selective pressure, indirectly promoting maintenance and co-transfer of MDR plasmids within bacterial populations.

**Fig 2:**
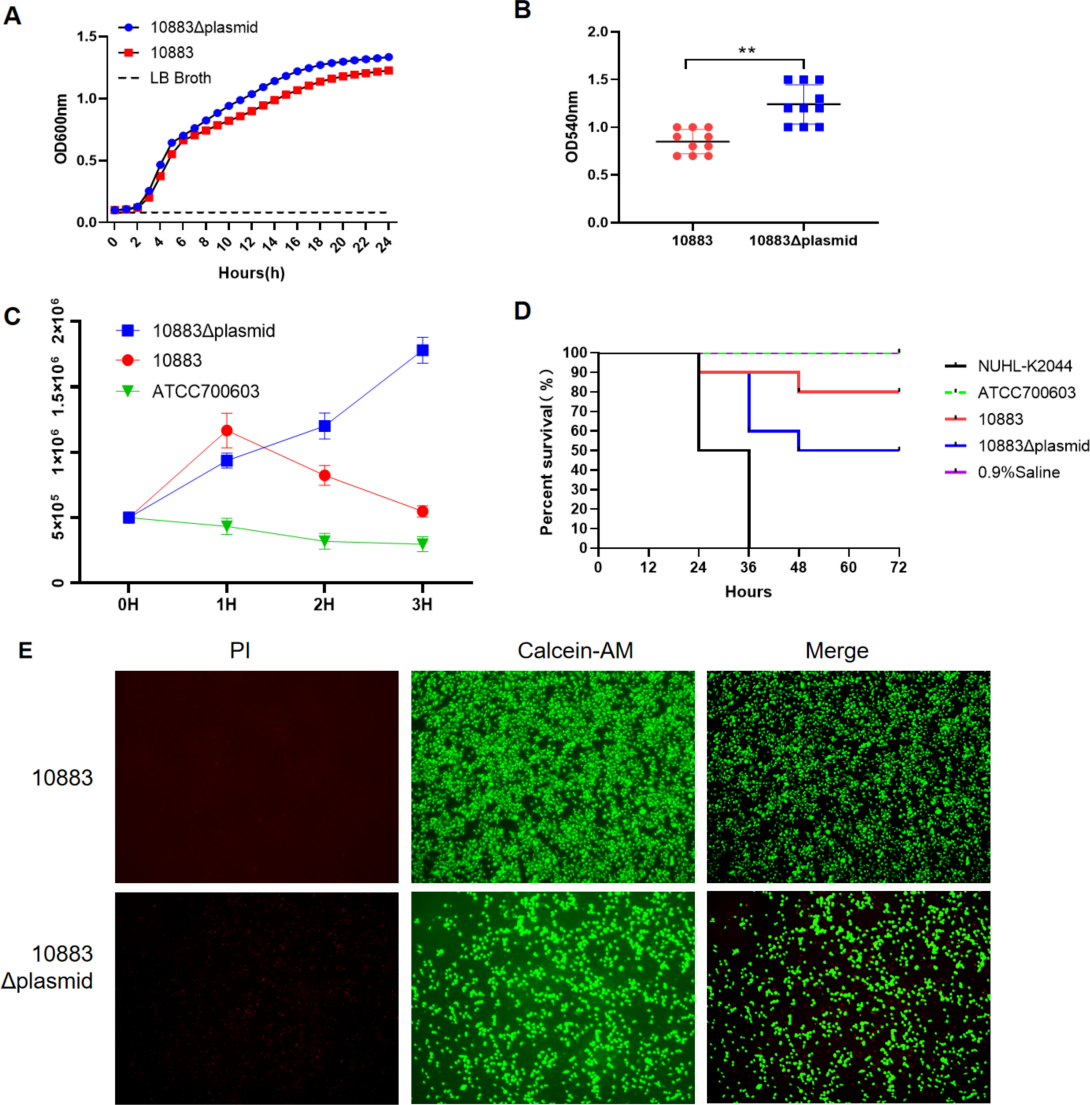
Impact of pKP10883-1 curing on growth (A), virulence phenotypes (**B–D**), and A549 cell viability after bacterial infection (E) in KP 10883. (**A**) Growth curves of the parental strain 10883 and its pKP10883-1-cured derivative 10883Δplasmid in LB broth at 37°C, showing no significant differences in growth dynamics. (**B**) Biofilm formation assessed by crystal violet staining. The plasmid-cured strain formed significantly more biofilm biomass than the parental strain (**P < 0.01). (**C**) Serum resistance assay in pooled normal human serum. The pKP10883-1-cured strain exhibited enhanced resistance, maintaining stable colony-forming unit (CFU) counts over 3 h, while the parental strain showed a marked reduction in viability. (**D**) Virulence assessment using the *G. mellonella* infection model. Larvae infected with the pKP10883-1-cured strain displayed a significantly higher mortality compared to those infected with the parental strain. A hypervirulent strain (NUHL-K2044) and a low-virulence control (ATCC 700603) were included as references. (**E**) A549 lung epithelial cells were infected with either the parental KP10883 strain or the pKP10883-1-cured derivative 10883Δplasmid and incubated in 50% pooled normal human serum for 3 h. Live and dead cells were stained with calcein-AM (green, viable cells) and propidium iodide (PI; red, dead cells) and imaged under a fluorescence microscope (Leica DMi8, 40× objective).

We next assessed the phenotypic contribution of pKP10883-1. Using SDS stress, we cured pKP10883-1 while retaining pKP10883-2, generating a 10883Δplasmid. In nutrient-replete LB, growth kinetics (Fig. 2A) were unchanged relative to the parental strain, implying minimal metabolic burden of pKP10883-1 under favorable conditions (8, 9). In contrast, antimicrobial susceptibility shifted profoundly: imipenem and meropenem minimum inhibitory concentrations (MICs) fell from ≥16/8 μg/mL to ≤0.25 µg/mL; those of ceftazidime and cefepime from ≥64/32 μg/mL to ≤0.12 µg/mL; and those of tigecycline fell from ≥8 µg/mL to ≤0.5 µg/mL . Restoration of susceptibility across β-lactams, carbapenems, fluoroquinolones, macrolides, sulfonamides, and tetracyclines confirms that pKP10883-1 is the principal driver of the MDR phenotype, consistent with the dominant effects of *bla*_NDM-1_ islands and *tmexCD-toprJ* efflux modules reported elsewhere (10–12).

Unexpectedly, plasmid loss unmasked stronger virulence-associated outputs. The 10883Δplasmid produced significantly denser biofilms (Fig. 2B, OD540 1.62 vs 0.89, *P* < 0.01), survived complement killing at grade 5–6 versus grade 2–3 for the parental strain (Fig. 2C), and caused greater lethality in *G. mellonella*, with 72-h survival <50% compared to ~80% for the parent (Fig. 2D), approaching—but not exceeding—hypervirulent (13, 14). Infection of A549 epithelial cells further showed increased cytotoxicity in the cured strain, evidenced by higher PI uptake and reduced calcein-AM fluorescence (Fig. 2E). These findings reveal a resistance-virulence trade-off in which the MDR megaplasmid suppresses chromosomal virulence programs, while its removal derepresses adhesion, serum resistance, and *in vivo* pathogenicity. Mechanistically, pKP10883-1 encodes putative H-NS-like nucleoid proteins and efflux-linked regulators capable of silencing horizontally acquired or stress-responsive loci. Derepression upon plasmid loss could enhance the expression of fimbrial operons, outer-membrane scaffolds (*ompA*), iron-capture systems, exopolysaccharide/capsule biosynthesis, and envelope-stress modules pathways repeatedly implicated in serum survival, biofilm maturation, host-cell damage, and lethal infection (15, 16). Although global transcriptomics and targeted mutagenesis will be required to pinpoint direct regulatory wiring, the observed phenotype is consistent with plasmid-encoded global regulators attenuating virulence to offset the costs of broad resistance or to reduce immune recognition in treated hosts.

Taken together, the close similarity between the resistance modules of pKP10883-1 and multiple well-characterized clinical plasmids suggests that its architecture arose through sequential acquisition and recombination events (17). The co-resident plasmid pKP10883-2 encodes stress-response and maintenance-related functions that may contribute to plasmid stability, although its specific role remains to be determined (18). Functionally, pKP10883-1 was sufficient to confer broad resistance to last-line antibiotics, and its removal restored susceptibility across all major drug classes. Importantly, curing pKP10883-1 also revealed increased biofilm formation, enhanced serum survival, greater epithelial cytotoxicity, and higher lethality in *G. mellonella*, consistent with a resistance-virulence trade-off in this isolate. Whether similar interactions occur across other *KpSC* backgrounds remains unclear and may depend on the strain-specific genomic context, antibiotic pressure, and host factors.

In summary, we report a *Kqss* strain co-producing *bla*_NDM-1_ and *tmexCD2-toprJ2* on a novel mosaic IncU megaplasmid, alongside a potential fitness-enhancing IncFII/FIB plasmid. The megaplasmid mediates extensive resistance to last-line agents but concurrently dampens virulence, whereas its removal restores susceptibility and unmasks stronger pathogenic phenotypes. These findings highlight the dynamic interplay between plasmid-mediated resistance and virulence in *Kqss* and emphasize the need for integrated resistance–virulence surveillance across the *KpSC* to better anticipate the clinical risks posed by emerging multidrug-resistant lineages.

## Data Availability

The Illumina sequences of all isolates are available in the NCBI database (accession number: PRJNA1261662).
